# Reply to: Letter to the Editor on the Paper: “Do the Clinical Practice Guidelines for Pediatric Dentistry Meet the Quality Standards?”

**DOI:** 10.1111/ipd.70015

**Published:** 2025-07-29

**Authors:** Rokaia Ahmed Elagami, Caroline Marino Laux, Tamara Kerber Tedesco, Thais Gimenez Cóvos, Mariana Minatel Braga, Fausto Medeiros Mendes, Maximiliano Sérgio Cenci, Daniela Prócida Raggio

**Affiliations:** ^1^ Department of Paediatric Dentistry School of Dentistry, University of São Paulo São Paulo Brazil; ^2^ Department of Dentistry Radboud University Medical Center, Research Institute for Medical Innovation Nijmegen the Netherlands

**Keywords:** community pediatric dentistry, dental education, restorative dentistry/dental materials

We appreciate the academic engagement expressed in the Letter to the Editor regarding our study titled “Do the Clinical Practice Guidelines for Pediatric Dentistry Meet the Quality Standards? A Meta‐Research and Quality Appraisal Using the AGREE II Tool”. We acknowledge our shared commitment to enhancing the reporting quality and methodological rigor of Clinical Practice Guidelines (CPGs) to ensure their validity, transparency, and reproducibility, ultimately benefiting researchers, stakeholders and clinicians. In this spirit, we welcome the opportunity to address key points related to the letter.

In response to the concerns raised in the Letter to the Editor regarding guideline selection, we would like to offer clarification. Our publication explicitly outlined the following inclusion criteria:

“A. Clinical practice guidelines that contain a “statement” or “guideline” or provide “recommendation” directed to paediatric dentists; and B. Guidelines that included at least one recommendation related to paediatric dentistry based on the literature and expert opinion”.

Both terms (CPG and Guideline) are commonly used interchangeably in the literature [1], as evidenced by sources such as the Institute for Quality and Efficiency in Health Care (IQWiG) in Germany (https://www.ncbi.nlm.nih.gov/books/NBK390308/), as well as the American Academy of Pediatric Dentistry (AAPD) in their reference manual overview (2022, 2024–2025) [2, 3], which outlines the CPG development process. According to the AAPD:

“The EBDC provides oversight and management of the CPG development process. The topic for each guideline is recommended by the EBDC and approved by the BOT”.

In this context, we included the six guidelines referenced in the Letter to the Editor, as they met our predefined inclusion criteria. These documents were explicitly labeled as “guidelines” in their titles and classified as “Clinical Practice Guidelines” in the footnotes of AAPD publications. We acknowledge that clear classification is essential to avoid confusion and enhance usability for the society, clinicians, and researchers. These guidelines remain publicly available on the AAPD website, continue to be cited in recent literature, and, in some cases, represent the only available guidance on their respective topics—highlighting the importance of assessing their methodological quality. Given their accessibility and influence on clinical decision‐making, evaluating their rigor is both necessary and valuable.

Additionally, we recognize the need for greater precision in the terminology of evidence‐based documents. This challenge has also been acknowledged in the medical field, as discussed by Aleksovska et al. (2021) [4] in their analysis of the distinctions between clinical practice guidelines and other types of evidence‐based documents.

Our study was explicitly designed as meta‐research, an interdisciplinary approach to researching research itself, with the aim of ensuring the transparency and methodological rigor of the evaluated documents—rather than assessing the validity of their recommendations. This intent was clearly stated in our final considerations, where we emphasized the need to raise awareness and improve future Clinical Practice Guidelines (CPGs). We underscored the importance of adhering to reporting guidelines in CPG development to enhance transparency and maximize their benefit to the broader community.

The Letter to the Editor raises concerns about the distinction between reporting quality and methodological rigor, particularly arguing that the AGREE II tool is intended to assess reporting rather than methodology. However, AGREE II explicitly includes Domain 3: Rigor of Development, which evaluates key methodological aspects such as systematic evidence searches, the strength of recommendations, criteria for selecting evidence, and stakeholder involvement. This domain ensures that guidelines adhere to robust methodological standards and helps mitigate potential biases.

Another point raised in the letter is that AGREE II does not assess the quality of evidence or the reliability of recommendations. We fully acknowledge this distinction and explicitly addressed it in the discussion section of our study. As we stated:

“The AGREE II instrument evaluates the methodological process and guideline structure but does not assess the content validity of the recommendations. Moreover, we adhered to AGREE II guidelines as closely as possible to ensure that our assessments were consistent and reliable. While our evaluation found that 25 guidelines did not meet AGREE II criteria for recommendation, this does not necessarily mean they lack sound clinical recommendations supported by reliable evidence. However, the absence of a structured reporting checklist in their development resulted in poor methodological reporting.”

Regarding your statement that *“the authors appropriately noted that there was improvement in AGREE II domains over time, especially in the past five years,”* we would like to clarify an important point. Our univariate linear regression analysis demonstrated improvement in all AGREE II domains except for Domain 4, with a decrease in scores as the years since publication increased. However, our model assessed the year since publication as a continuous variable rather than categorizing data into predefined time intervals, such as the past 5 years. Therefore, the observed improvement was not specifically limited to this timeframe but rather reflected a general trend over time.

The Letter to the Editor raised concerns regarding the inclusion of older guidelines published before 2016, noting that many predated the development of the AGREE reporting checklist. We acknowledge this point and have already addressed it in our discussion:

“We recognize that many of the included guidelines were published before the launch of the AGREE II tool in 2010. However, the original AGREE tool had been validated and available since 2001, allowing for retrospective assessments of earlier guidelines. To mitigate this limitation, we included only the most recent version whenever a guideline had been updated. Therefore, we believe that the benefit of achieving comprehensive coverage of pediatric dentistry guidelines outweighs the potential limitation of including a single guideline from the year 2000.”

Furthermore, while the AGREE reporting checklist was introduced in 2016 [5] to improve guideline reporting based on the AGREE tool—an internationally accepted standard for evaluating the methodological quality of CPGs since 2001—other reporting checklists and guidance documents were available and followed by some of the CPGs included in our study. Notably, organizations such as:

- The National Institute for Health and Care Excellence (NICE) (guidelines manual, 2009),
- The Scottish Intercollegiate Guidelines Network (SIGN) (guideline development handbook, since 1999), and
- The World Health Organization (WHO) (guideline development manual, since 2003).

had long established rigorous methodologies for guideline development. These frameworks adhere to the same core principles of transparency, methodological rigor, and evidence‐based practice that are recognized in modern CPG development [6].

Additionally, the Letter to the Editor questioned the clarity of reporting regarding the 15 guidelines older than 10 years and their respective organizations. However, this information is explicitly detailed in File S3 in our primary publication, which provides a comprehensive table classifying all included guidelines by organization, along with their titles and publication years. This ensures that guidelines older than 10 years can be clearly identified.

We agree with the authors of the Letter to the Editor that CPGs are generally recommended to be updated every 5 years, and we acknowledge that a direct comparison between guidelines published in the last 5 years and earlier ones could be a valuable avenue for future research. However, we also recognize that updates may not always be necessary in the absence of substantial new evidence.

The primary objective of our study was to evaluate the reporting quality of the most recent and available CPGs, while our secondary objective was to explore potential factors associated with reporting quality. One such factor was years since publication, which we calculated as the difference between the guideline's publication year and the year of our search strategy. Specifically, we examined whether reporting quality—both in individual AGREE II domains and overall scores—had improved over time using regression analysis. We appreciate the suggestion to report Odds Ratios (ORs) with Confidence Intervals (CIs) instead of standard errors and will consider this approach in future analyses.

We value this academic exchange and the opportunity to clarify our methodology and findings. Importantly, we acknowledge the authors of the Letter to the Editor for recognizing the critical need to evaluate both the reporting quality and methodological rigor of CPGs. To strengthen future guideline development, we emphasize the importance of adopting appropriate reporting checklists to enhance transparency and reliability. Additionally, we advocate for greater clarity in the classification and terminology of CPGs, ensuring that only the most up‐to‐date versions remain available on organizational websites. Such refinements are essential for minimizing confusion among clinicians and researchers, ultimately supporting more effective, evidence‐based decision‐making in pediatric dentistry.

Perhaps this discussion presents an opportunity to reflect on and work toward standardizing terminology on all documents available from the associations, ensuring that policymakers, clinicians, researchers, and ultimately patients can fully understand and benefit from the extensive efforts dedicated to their development.

## Author Contributions

R.A.E., F.M.M. drafted the manuscript; D.P.R. critically reviewed and edited the letter. All the authors approved the final version of the manuscript.

## Conflicts of Interest

The authors declare no conflicts of interest.

## References

[1] S. H. Woolf , R. Grol , A. Hutchinson , M. Eccles , and J. Grimshaw , “Clinical Guidelines: Potential Benefits, Limitations, and Harms of Clinical Guidelines,” BMJ (Clinical Research Ed.) 318, no. 7182 (1999): 527–530, 10.1136/bmj.318.7182.527. PMC1114973 10024268

[2] American Academy of Pediatric Dentistry , “Overview,” in The Reference Manual of Pediatric Dentistry (American Academy of Pediatric Dentistry, 2022), 7–9.

[3] American Academy of Pediatric Dentistry , The Reference Manual of Pediatric Dentistry (American Academy of Pediatric Dentistry, 2024), 7–9.

[4] K. Aleksovska , C. L. A. Bassetti , T. Berger , et al., “Guidelines Should Be Guidelines: Time to Leave the Terms “Consensus” and “Position” for Other Purposes,” European Journal of Neurology 28 (2021): 2461–2466, 10.1111/ene.14933. 34022083

[5] “The AGREE Reporting Checklist: A Tool to Improve Reporting of Clinical Practice Guidelines,” BMJ 354 (2016): i4852, 10.1136/bmj.i4852. 27600405 PMC5012748

[6] A. R. Gagliardi and M. C. Brouwers , “Integrating Guideline Development and Implementation: Analysis of Guideline Development Manual Instructions for Generating Implementation Advice,” Implementation Science 7 (2012): 67, 10.1186/1748-5908-7-67. 22824094 PMC3457906

